# Photon-counting CT for coronary stent evaluation: OCT-validated case of severe in-stent restenosis

**DOI:** 10.1007/s10554-025-03484-w

**Published:** 2025-07-31

**Authors:** Lili Száraz, Péter Kulyassa, Pál Maurovich-Horvat, Bálint Szilveszter

**Affiliations:** 1 Department of Radiology, Clinic for Medical Imaging, Semmelweis University, Budapest, Hungary; 2 Semmelweis University Heart and Vascular Center, Budapest, Hungary

**Keywords:** Photon-counting detector CT, Optical coherence tomography, Coronary artery stent, In-stent-restenosis, Plaque characterization

## Abstract

Photon-counting detector CT (PCD-CT) has the potential to transform stent assessment by improving visualization of stent patency and in-stent restenosis (ISR) characterization utilizing improved spatial resolution. This report presents a case of ISR evaluated using PCD-CT, with direct comparison to optical coherence tomography (OCT), as the current gold standard.

**Supplementary Information:**

The online version contains supplementary material available at 10.1007/s10554-025-03484-w.

## Case summary

A 71-year-old male with stable chest pain and a history of left anterior descending coronary artery stent implantation. Coronary CT angiography revealed CAD-RADS 4B/P4/HRP/S, including (1) pre-stent stenosis, caused by a predominantly non-calcified plaque, and (2) severe ISR with fibro-fatty lesions.

OCT confirmed the presence of ISR, with circumferential, heterogeneous neoatherosclerotic lesions with lipid arcs, micro calcification and translucent fibrotic tissue. The OCT and PCD-CT derived measurements showed strong correlation, with minor differences: lumen area of 8.0 mm^2^ vs. 8.2 mm^2^; stent diameter: min. of 3.3 mm vs. 3.5 mm; max. of 3.9 mm vs. 3.7 mm, respectively. Furthermore, the matched stent segments showed excellent agreement on cross-sectional tissue characterization [1].

**Fig. 1:**
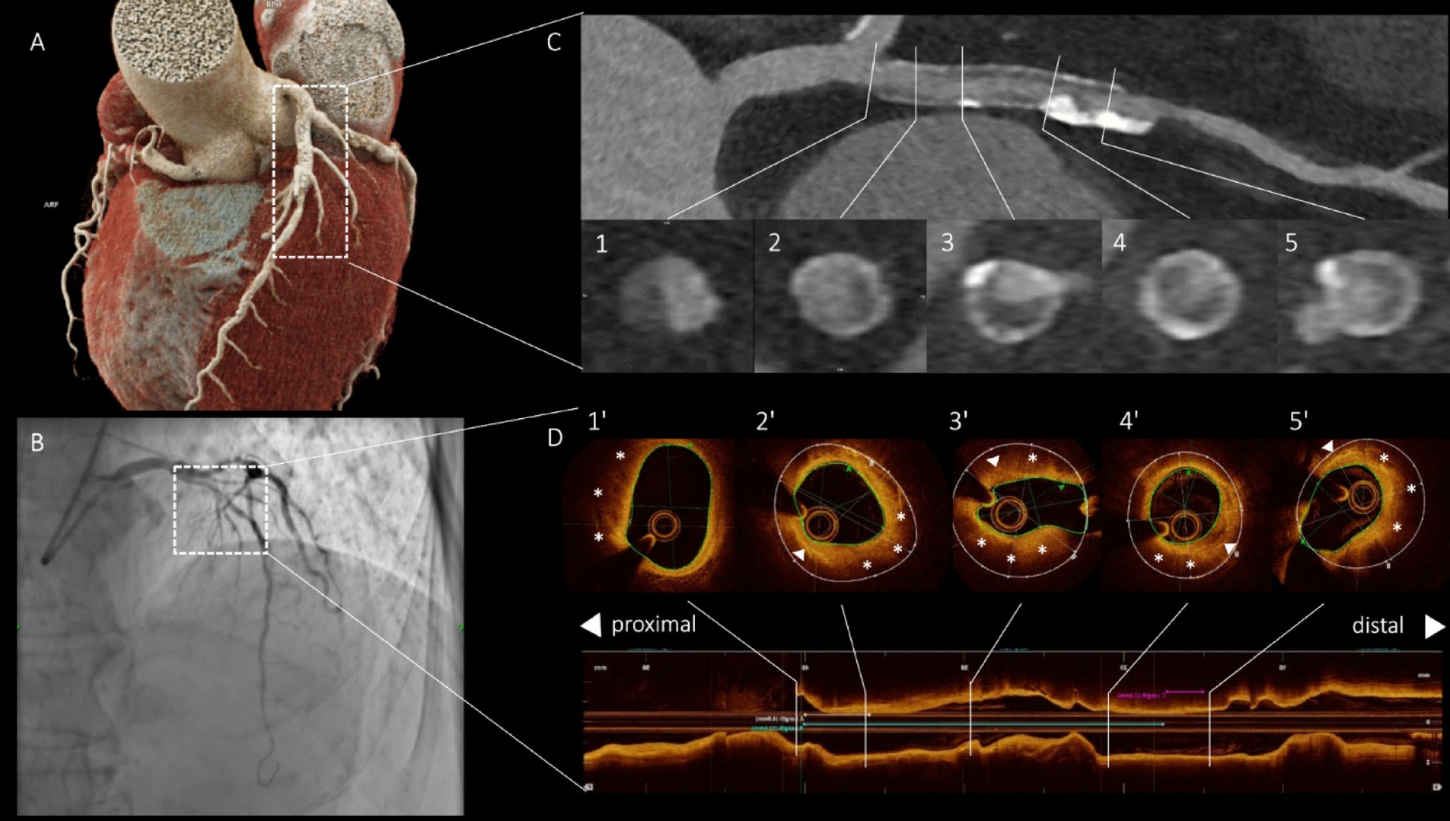
Co-registration of Coronary CT Angiography and Optical Coherence Tomography Images Across Proximal to Distal Vessel Segments. Panels 1–5 (CTA) and 1′–5′ (OCT) illustrate matched coronary segments from proximal to distal regions. Asterisks indicate lipid arcs, while arrows highlight microcalcifications

In this case, PCD-CT enabled ultrahigh-resolution visualization of the stent and ISR characterization, closely matching the gold standard OCT findings. These results underscore the value and the clinical potential of PCD-CT as a promising non-invasive tool for assessing stent patency (Fig. 1).

## Supplementary Information

Below is the link to the electronic supplementary material.

Supplementary Material 1

## Data Availability

No datasets were generated or analysed during the current study.
