# Supplementary material for: Photon-counting CT for coronary stent evaluation: OCT-validated case of severe in-stent restenosis
Source: Int J Cardiovasc Imaging. 2025 Jul 31;41(9):1845–6. doi: 10.1007/s10554-025-03484-w (PMC12405332; doi:10.1007/s10554-025-03484-w)
Supplement: Supplementary file 1 — Supplementary Material 1 [file 10554_2025_3484_MOESM1_ESM.docx]

**Supplementary**

Table 1: Examination protocol

| Scanner | NAEOTOM Alpha.Peak (Siemens Healthineers, Forchheim, Germany) |
| --- | --- |
| Scan mode | UHR mode (Quantum HD Cardiac) |
| Tube voltage | 120 kV |
| Effective mAs | 65 mAs |
| IQ level | 64 |
| Dose modulation | CARE Dose4D |
| CTDIvol | 21.4 mGy |
| DLP | 276 mGy*cm |
| Rotation time | 0.25 s |
| Pitch | 0.18 |
| Slice collimation | 120 x 0.2 mm |
| Slice width | 0.2 mm |
| Reconstruction increment | 0.2 mm |
| Reconstruction kernel | Bv60, QIR level of 3 |
| Reconstruction matrix | 512 x 512 |
| Heart rate | 48 – 49 bpm |
| Contrast | Iomeron 400 mg/mL |
| Volume | Four-phasic injection protocol:   - 10 ml pure Saline - 83 mL pure CM - 30 mL (40% CM, 60% Saline) - 50 mL pure Saline |
| Flow rate | 5 mL/s |
| Start delay | Bolus tracking triggered at 150 HU in the left atrium + 4 sec. |
